# Book Reviews

**Published:** 1893-10

**Authors:** 


### Book Reviews.

A TEXT-BOOK OF THE THEORY AND PRACTICE OF MEDICINE. By American teachers. Edited by WILLIAM PEPPER. M. D., LL. D., Provost and Professor of the Theory and Practice of Medicine and of Clinical Medicine in the University of Pennsylvania. In two volumes; illustrated. Vol. I. Large octavo, pp. 909. Philadelphia: W. B. Saunders, 913 Walnut street. 1893.

The list of authors who have written for this work comprises the names of some of our most eminent and admired teachers,—men who have for many years led the ranks in medicine. The names of those contributing to this volume are Billings, Osler, Pepper, Thompson, Whittaker, and Wood.

John S. Billings contributes an admirable article upon Hygiene, which occupies first place in the volume and sets forth the great principles of hygiene, supported by statistics, clear argument, and calm reasoning. We consider it a noticeable step in advance that this sound and elaborate article upon hygiene should fill the first pages of a modern text-book of medicine.

William Pepper, among other articles, contributes one of especial value upon Typhoid Fever, in which eighteen pages are devoted to treatment. We are glad to see that Dr. Pepper is opposed to the use of semi-solid or solid food during the course of typhoid fever; and, while he says some excellent observers sanction its use, he has repeatedly observed harmful effects from its administration. The tenor of Dr. Pepper's remarks upon the administration of alcohol in typhoid fever indicates that he deprecates the use of alcohol simply because the diagnosis of typhoid fever is admissible, and he evidently advises the use of alcoholic stimulants only when there is profound constitutional disturbance, while in mild cases which are progressing favorably alcohol should be withheld. In the consideration of the temperature of typhoid fever, Dr. Pepper says that the high mortality in this disease comes, directly or indirectly, from persistent pronounced pyrexia, and he advocates the use of cold baths, according to the Brand method, in order that the temperature may be kept below 102.5° Fahrenheit. Other methods of application of cold to the surface of the body, in typhoid fever, for the reduction of temperature, are described; but, happily, Dr. Pepper places the highest value upon the Brand treatment, the details of which he carefully gives. We do not think, however, that Dr. Pepper sufficiently emphasizes the beneficial effects of the cold baths upon the nervous system, the circulation, the digestive apparatus, and the general tone of the patient; nor does he lay enough stress upon the fact that complications are rendered less frequent and less serious under the bath treatment; nor does he show how medication may be reduced to a minimum or entirely dispensed with, and how the therapeutics of typhoid fever may be greatly simplified, so as to consist merely of the regular administration of milk, attention to the hygienic surroundings, good nursing, and the cold plunges. From the pen of so able a man as Dr. Pepper, we should be pleased to see a plea for simplified therapeutics in typhoid fever. As regards the administration of intestinal antiseptics in typhoid fever, more caution and discrimination should be advised, as it is with a delusive hope of affecting the intestinal lesions and the specific bacteria of the disease that so many physicians give, as we have often had occasion to notice, numerous doses of the most incompatible and disgusting combinations. Too free medication in typhoid fever is to be deprecated as urgently as the cold plunge treatment is to be advocated.

James T. Whittaker writes well-arranged and concise articles upon the Exanthemata, which admit of easy reference, and are valuable alike for practitioner and student. The chapters written by W. Gilman Thompson are noticeable for the careful and detailed account of the specific agents in the causation of diphtheria and the malarial fevers. These chapters are otherwise well up to date, and are worthy of careful reading.

H. C. Wood writes the section upon Diseases of the Nervous System in his familiar felicitous style, which makes delightful reading of this portion of the volume. His articles upon Neurasthenia and Hysteria are concise and to the point, although the failure to mention eye-strain as a causative element in these affections renders their discussion far from complete. The correction of ocular errors, refractive or muscular, has done so much toward relieving hysteria and neurasthenia that it is no longer a subject to scoff at, but one which should engage our earnest attention.

Among other excellent contributions to this volume, William Osler writes a valuable chapter upon Vaso-motor and Trophic Disorders, in which are discussed Raynaud's Disease, Angio-neurotic Edema, and Acromegaly, affections the literature of which has been heretofore found chiefly in the journals. This volume has more than usual merit, in that it is composed of the most advanced writings of some of the ablest among our American physicians, and we should be proud of it as a purely American production and as an effort that reaches a high standard. A. A. J.

---

HUMAN EMBRYOLOGY. By CHARLES SEDGWICK MINOT, Professor of Histology and Human Embryology, Harvard Medical School, Boston. 463 illustrations. New York: William Wood & Co. 1892.

Professor Minot has presented, in this volume of some 800 pages, an exhaustive treatise of the subject. It appeals most strongly to the advanced student of embryology, but even the average medical reader, who has only the most rudimentary knowledge of the subject, can appreciate the magnitude of the work, and the years of earnest, persistent labor that have been required to complete it.

The introductory chapters treat of the Anatomy and Physiology of the Uterus, with special reference to the histology of it's mucosa, and the transitions which the latter undergoes during functional activity. The formation, minute structure, and function of the deciduæ, are discussed in detail. The author shows that the functions of menstruation and gestation are essentially homologous—the former being simply less prolonged and intensified,—in fact, a mimic labor.

Part I., on the Genital Products, contains five chapters, the first two of which are devoted to the life history of the spermatazoon and ovum, respectively. The third and fourth, to ovulation and impregnation, and the last, to a discussion of the theories of sex and heredity.

It is from a careful study of the genesis of the sexual elements that he obtains the data from which his theory of the nature of sexuality is deduced. He says: "This hypothesis is based upon three categories of facts: First, sexual reproduction is effected by the union of a male and female element, which produces a cell; this cell is, therefore, hermaphroditic, or perhaps one should say, asexual or neuter, since it is neither male nor female; second, when the cell which gives rise to the female element matures into an ovum, it undergoes a remarkable process of unequal division, known as the extrusion of the polar globules; in other words, the cell divides into three bodies—(*a*) two polar globules, (*b*) a single female element—in some cases the polar globules divide further; third, when a cell divides into male elements, there remains one cell which does not form a spermatazoon; in mammals it is probable that the parent cell divides into three cells, one of which (*b*) remains to form the base of Sertoli's column, and two of which (*a*) subdivide further to produce the spermatoblasts, and, ultimately, the spermatozoa."

Assuming that this view of spermatogenesis is true, the author shows that in each cell both sexes are potentially present—that in order to produce sexual elements (genoblasts), the cell divides into its several parts; in the case of the egg-cell, the male polar globules are thrown off, leaving the female ovum. In the case of the sperm-cell, the male spermatoblasts, which are supposed to be analogous to the polar globules, multiply considerably, and their descendants give rise to the male elements, while the parent cell atrophies. In order to make a complete cell, which will be capable of developing into a fetus, the union of these sexual elements is necessary. According to this view, parthenogenesis is only an extreme case of asexual reproduction, and in nowise the development of a female element without impregnation.

The theories of heredity are briefly stated, and the author's views thus summarized: "The child is like its parents, because its organization is regulated by not merely similar, but by some of the same chromatine (nuclear substance) as that of its parents."

Part II., on the Germ Layers, includes (Chapter IV.), Segmentation (Chapter V.), Concresence, the law of which is stated on page 125: "The vertebrate primitive axis is formed by the growing together in the axial line of the future embryo of the two halves of the ectental line" (Chapter VI.). The Mesoderm and the Coelom, in which the various theories of that much disputed question, the origin of the mesoderm, are thoroughly discussed, and the conclusion drawn that it is derived from the entoderm (Chapter VII.), General Remarks on the Germ Layers.

Part III., on the Embryo, includes Chapters VIII. to XIII.; on the Medullary Groove, Notochord, and Neurenteric Canals; Division of the Coelom; Origin of the Blood, Blood-vessels, and Heart; Origin of the Urogenital System; The Archenteron and the Gill Clefts; The Germinal Area; The Embryo, and its Appendages.

Parts III. and IV. are replete with interesting facts, a detailed review of which, we regret, space will not permit.

In Part V., an excellent account is given of the growth and development of the fetus, its appendages, organs, and special systems. This is the largest part of the book, covering over 300 pages.

We can speak none but words of praise for this valuable contribution to the literature of embryology. The author's statements are clear and concise, and a thoroughly scientific spirit is manifested throughout the entire treatise.

The drawings from microscopical sections call for special commendation. Only those who have made such drawings can appreciate the labor involved.

Many authors have been quoted, and full credit is given in the remarkable bibliographical reference list, which is appended.

The publishers have done their part well, and the book should have a wide circulation. E. P. L.

---

HYGIENE AND PUBLIC HEALTH. By LOUIS C. PARKES, M. D., D. P. H., London University. Fellow of the Sanitary Institute, and Member of the Board of Examiners; Lecturer on Public Health at St. George's Hospital Medical School; Medical Officer of Health and Public Analysis for the parish of Chelsea. Third edition, with illustrations. Price, $2.75. Philadelphia: P. Blakiston, Son & Co., 1012 Walnut street. 1892.

The study of the science of hygiene and public health has progressed in a remarkable degree within the last decade. It has come to be a necessary part of the equipment of every physician, whether he is a specialist or a general practitioner of medicine and surgery, or whether he is simply a laboratory teacher, to be familiar with the essential subjects treated under this general head. Every physician, nowadays, is asked questions that come within the province of the sanitarian and public health scientist, and he must be ready to give an intelligent answer when this information is sought. The people are not slow to understand that it is more important to prevent disease than to cure it, and even those who are only moderately informed on science in general are apt to have some information on the prevention of disease, through care of the person, house sanitation, and good drainage.

This work has passed through two editions within a comparatively short time, and now presents itself in an admirable dress, with some additions and improvements, and a thorough revision.

The subject of Smoke Prevention by Mechanical Appliances, of Weather Observations, of Cyclonic Systems, and of Epidemic Influenza, have been newly introduced, while the article on Diphtheria has been entirely re-written, and other chapters, dealing with Etiology and Bacteriology, have been brought up to date.

We are pleased to give endorsement to such an admirable work, for it must be so regarded, alike in matter, method, and make-up. It has a very complete index, which we desire to especially commend.

---

Diseases of the Kidneys and Bladder. A Text-book for Students of Medicine. By W. F. McNutt, M. D., M. R. C. S., Ed., L. R. C. P., Ed., Professor of the Principles and Practice of Medicine, University of California; Professor of the Diseases of the Kidneys and Heart, Post-Graduate Department of the University of California; Consulting Physician and Surgeon to St. Mary's Hospital, and to the Children's Hospital; Member of the International Medical Congress, of the American Medical Association, and of the California State Medical Society; President of the California Gynecological Society; Ex-Acting Assistant Surgeon United States Navy, etc. Philadelphia: J. B. Lippincott Co. 1893.

This work begins by giving the anatomy and physiology of the kidneys, including anomalies of position, form, and number. In its second section, Diseases of the Kidney are taken up, the first being nephralgia, or neuralgia of the kidneys. There is scarcely any organ or section of the body which may not be the seat of this distressing symptom, for, in most instances, neuralgia is a symptom of some significance relating to structural or functional disease. It is especially so in the kidneys, where calculi furnish the chief cause for pain in the organ.

Hyperemia, Hematuria, Anemia, Disease of the Renal Blood-vessels, and, next, the Urine are taken up in their natural order. The chapter on the Urine is an exceedingly interesting one, and deserves careful study. Casts of the Uriniferous Tubules, Albumen, and Nephritis in its various forms, are then discussed in the order named.

The author is very clear in his treatment of the subjects grouped under the general head Nephritis, and has illustrated its pathology with a few cuts, and these, it seems to us, might have been increased in number with much profit. Uremia is next taken up, then Degenerations and New Growths.

Section III. considers Diseases of the Pelvis of the Kidneys, including the ureters, and finally, in the next chapter, the Surgical Treatment of the Kidneys is dwelt upon.

Section IV. considers Diseases of the Bladder, which is a department of medicine requiring more careful attention by the general practitioner than it usually receives. He will here find carefully considered instructions that will aid him in the diagnosis and treatment of these sometimes very subtle conditions.

In Section V., Diabetes is treated upon, and the volume closes with an excellent index.

We commend it to the careful consideration of the general practitioner as well as the genito-urinary specialist.

---

CARDIAC OUTLINES FOR CLINICAL CLERKS AND PRACTITIONERS, AND FIRST PRINCIPLES IN THE PHYSICAL EXAMINATION OF THE HEART, FOR THE BEGINNER. By WILLIAM EWART, M. D., Cantab., F. R. C. P., Physician to St. George's Hospital; Clinical Lecturer and Teacher of Practical Medicine in the Medical School; Physician to the Belgrave Hospital for Children; Additional Examiner, in 1891, for the third M. B. of the University of Cambridge; late Assistant Physician and Pathologist to the Brompton Hospital for Consumption and Diseases of the Chest. With sixty-two illustrations. New York: G. P. Putnam's Sons, 27 West Twenty-third street. London: The Knickerbocker Press, 24 Bedford street, Strand. 1892.

The author of this monograph states in his preface that he indited the work with a three-fold object, namely: To help the beginner through his first difficulties; to encourage the clinical clerk in the cultivation of a graphic method as a means to thoroughness and accuracy; and to place at the disposal of the practitioner a way and method of adequately recording important clinical observations.

A careful examination of the work indicates that Mr. Ewart has admirably succeeded on the three lines laid out. The importance of stating clinically the location of the heart in health and disease cannot be over-estimated, for disease of this important organ must be early diagnosticated, if its multifarious diseases are to be relieved. Anatomical accuracy is essential as a beginning to diagnosis.

We commend this little work to the student of clinical accuracy, and to practitioners of medicine, as a valuable guide in their researches and investigations.

---

LESSONS IN PHYSICAL DIAGNOSIS. By ALFRED L. LOOMIS, M. D., LL.D., Professor of the Practice of Medicine and Pathology in the University of the City of New York. Tenth edition, revised and enlarged. Octavo; illustrations, some in color; 240 pages, extra muslin; price, $3.00. New York: William Wood & Co.

The tenth edition of this volume requires but very little notice at our hands, beyond the mere announcement of the fact that this distinguished author and teacher has kept his work well to the fore in the point of progress. It has been thoroughly revised, and such corrections and additions have been made as seemed to him necessary to make it fully abreast of the present moment.

Dr. Loomis says, in a prefatory note, that the section on the physical action of the heart, and the lesson on the examination of the urine, have been entirely re-written, and that a new lesson on clinical microscopy has been added.

We commend the lesson on the examination of the urine to every student and practitioner interested—and who is not ?—as worthy of the most careful study. It is a subject second to none in importance, for through the examination of the urine often has been found the key to many an obscure malady; but this work must be done carefully, and not in the usual slipshod manner. Dr. Loomis is especially strong in reference to the diagnosis of cardiac diseases, and has long been the accepted authority in this branch of internal medicine. Here, again, his teachings deserve careful reading, since they furnish a safe guide in this important field.

The book is handsomely printed, and contains many useful illustrations.

---

Napheys' Modern Therapeutics. Medical and Surgical, including the Diseases of Women and Children. A compendium of recent formula and therapeutical directions from the practice of eminent contemporary physicians, American and foreign. Ninth edition, revised and enlarged. Volume II. General Surgery, Gynecology, and Obstetrics. By Allen J. Smith, M. D., Professor of Pathology, University of Texas, Galveston; late Assistant Demonstrator of Morbid Anatomy and Pathological Histology, and Lecturer on Urinology, University of Pennsylvania, and J. Aubrey Davis, M. D., Assistant Demonstrator of Obstetrics, University of Pennsylvania; Assistant Physician to Home for Crippled Children, Philadelphia. Large octavo volume. Pp. 19–1112. Price, $6.00 Philadelphia: P. Blakiston, Son & Co., 1012 Walnut street. 1893.

The second volume of this important work is constructed on similar lines to that of Volume I., which we noticed in the issue of the Journal for December, 1892. It is scarcely within the province of the reviewer to go beyond the mere statement that this work is one of the most practical of its kind, and must necessarily prove useful in a marked degree to the general practitioner of medicine. This is its appropriate field, in our opinion, for here is recorded, briefly, such practical therapeutical points as will serve to aid him in the treatment of disease. Besides the many useful formulæ that the volume contains, there are numerous clinical memoranda and suggestions which are not usually found in therapeutical treatises.

The ninth edition of a work usually bespeaks its popularity, and we know of none that has become more so in twenty-two years than Napheys' Modern Therapeutics.

FERMENTATION, INFECTION, AND IMMUNITY. A New Theory of these Processes, which Unifies their Primary Causation and Places the Explanation of their Phenomena in Chemistry, Biology, and the Dynamics of Molecular Physics. By J. W. MCLAUGHLIN, M. D. Pp. 240. Austin, Texas : Eugene Von Boeckman, printer.

This so-called new theory is no new theory at all, but an elaboration of the old physical theory of Liebig. Dr. McLaughlin, however, rids the old theory of much of its crudeness, and presents the physical theory in a manner which is most intensely interesting reading.

We congratulate the author upon having given the scientist a very interesting description of fermentation, and having made a very laudable attempt to explain the same by means of molecular physics.

The theory of fermentation, in a nut-shell, as presented by Dr. McLaughlin, is that yeast (saccharomyces cerevisiæ, for example,) has a certain molecular motion as regards wave length and period of time; that the sugar with which it comes in contact, and in which it produces the phenomena known as fermentation, has a molecular motion which coincides, or nearly coincides, with the wave length and period of time of the yeast cell. Now, the constant beating of the yeast cell against the molecule of sugar causes the sugar molecule to be resolved into its atomic constituents, and these atoms re-arrange themselves under the formation of ethyl alcohol, carbon, dioxide, propyl, isopropyl, and butyl alcohols, succinnic acid, glycerine, etc.

The theory is a grand one, but why, in the fermentation of dextrose by saccharomyces cerevisiæ, ethyl acohol should be formed in by far the largest quantity, is not explained. Viewed from the standpoint of chemical possibility, the molecule of dextrose might just as well re-arrange its atoms into methyl alcohol, propyl alcohol, and carbon dioxide, as into the products which we know are produced.

On the other hand, take the unorganized ferments. Do they produce their specific effect by means of bombardment ? Does diastose, for instance, train its dynamite guns upon the starch molecule and resolve it into its atomic elements ? If such is the case, then hydrochloric, sulphuric, and other acids must convert the starch molecule into sugar by a similar process. In regard to the unorganized ferments, we do not know but that they combine with the molecule upon which they are to act, and that this combination is again decomposed. Let us not forget the mystery which long surrounded the formation of ether from alcohol and sulphuric acid. "Contact action" explained, for a great many years, a process which we know today is simply synthesis and decomposition.

Another point in the theory, which appears very weak to us, is the action of one specific form of yeast upon sugars which are physically and chemically different. For example, why should saccharomyces cerevisiæ produce the same products from dextrose and levulose, two sugars physically different, or from maltose, a sugar different, both chemically and physically, from the two just mentioned?

The theory advanced, while very plausible, fails to explain many peculiarities met with in actual practice. J. A. M.

---

DISEASES OF THE HEART, LUNGS, AND KIDNEYS. By N. S. DAVIS, Jr., A. M., M. D., Professor of Principles and Practice of Medicine, Chicago Medical College: Physician to Mercy Hospital, etc., etc. Philadelphia and London: F. A. Davis & Co., Publishers. 1892.

The author states in the preface that this volume comprises a part of the topics of his lectures before the students of the Chicago Medical College, and this presumably is the reason for their appearance in book form. The various affections of these organs are treated carefully and logically, but contain nothing new or original, and nothing that cannot be found in any good book on practice. The author treats the various forms of disease of these organs very carefully and conscientiously, giving formulas and special directions wherever convenient. The binding is similar to the other volumes of the ready reference series. W. C. K.

---

MANUAL OF CHEMISTRY. A Guide to Lectures and Laboratory Work for Beginners in Chemistry. A Text-Book Specially Adapted for Students of Medicine and Pharmacy. By W. SIMON, Ph. D., M. D., Professor of Chemistry and Toxicology in the College of Physicians and Surgeons; Professor of Chemistry and Analytical Chemistry in the Maryland College of Pharmacy, Baltimore, Md. Fourth edition, thoroughly revised, with forty-four illustrations and seven colored plates, representing fifty-six chemical reactions. Pp. vii.—493. Philadelphia: Lea Brothers & Co. 1893.

This work is by no means a new one to the profession, and its popularity is evidenced by the call for a fourth edition in a comparatively short time. The author has brought it down to the present status of chemical science. The colored plates used for the purpose of illustrating the different precipitates is a great aid to the student, and highly commendable.

This work is written in a clear, concise style, and imparts to the student who will study it carefully, an excellent outline of the science.

The typography and make-up of the book is in the usual good taste which is characteristic of this firm. J. A. M.

### BOOKS RECEIVED.

A Manual of Medical Treatment or Clinical Therapeutics. By I. Burney Yeo, M. D., F. R. C. P., Professor of Therapeutics in King's College, London. In two 12mo volumes, containing 1275 pages, with illustrations. Complete work, cloth, $5.50. Philadelphia: Lea Brothers & Co. 1893.

The Throat and Nose, and Their Diseases. By Lennox Browne, F. R. C. S. E , Senior Physician to the Central London Throat and Ear Hospital. Fourth and enlarged edition. In one imperial octavo volume of about 750 pages, with 120 illustrations in color, and 235 engravings on wood. Cloth, $6.50. Philadelphia: Lea Brothers & Co. 1893.

A Manual of Diseases of the Ear. By George P. Field, M. R. C. S., Aural Surgeon and Lecturer on Aural Surgery, St. Mary's Hospital Medical School, London. In one octavo volume of 391 pages, with seventy-three engravings and twenty-one colored plates. Cloth, $3.75. Philadelphia: Lea Brothers & Co. 1893.

Annual of the Universal Medical Sciences. A Yearly Report of the Progress of the General Sanitary Sciences throughout the World. Edited by Charles E. Sajous, M. D., and seventy associate editors, assisted by over two hundred corresponding editors, collaborators, and correspondents. Illustrated with chromo-lithographs, engravings, and maps. Five volumes. The F. A. Davis Company, Publishers, Philadelphia, New York, Chicago, and London. Australian Agency: Melbourne, Victoria. 1893.

The Art of Preserving Health by Preventing Disease. Outlines of Practical Hygiene, adapted to American Conditions. By C. Gilman Currier, M. D., Visiting Physician to the New York City Hospitals; Fellow of the New York Academy of Medicine; Member of the New York Pathological Society; Member of the American Medical Association, etc., etc. One large octavo volume, 468 pages, illustrated, $2.75. New York: E. B. Treat, 5 Cooper Union. 1893.

A Chapter on Cholera, for Lay Readers. History, Symptoms, Prevention, and Treatment of the Disease. By Walter Vought, Ph.B., M. D., Medical Director and Physician-in-Charge of the Fire Island Quarantine Station, Port of New York; Fellow of the New York Academy of Medicine, etc. Illustrated with colored plates and wood engravings. In one small 12mo volume, 110 pages. Price, 75 cents net. Philadelphia: The F. A. Davis Co., Publishers, 1914 and 1916 Cherry street.

The Pharmacopeia of the United States of America. Seventh decennial revision (1890). By authority of the National Convention for Revising the Pharmacopeia, held at Washington, A. D. 1890. Official for January 1, 1894. Published by the Committee on Revision. Octavo pp. l.—602. Philadelphia: J. B. Lippincott Company, Printers and Binders. P. Blakiston, Son & Co., Agents. 1893.

A Treatise on Ophthalmology, for the General Practitioner. By Adolph Alt, M. D. Second edition. Revised and enlarged, with 140 illustrations, 8vo, pp. xvi. – 330. St. Louis: J. H. Chambers & Co. 1893.

Transactions of the Texas State Medical Association. Twenty-fifth Annual Session, held at Galveston, May 2, 3, 4, and 5, 1893. Edited by H. A. West, M. D., Secretary. Octavo volume, pp. 448. Galveston: Knapp Brothers, Printers and Publishers. 1893.

Transactions of the Association of American Physicians. Eighth Session, held at Washington. D. C., May 30–31, and June 1, 1893. Volume VIII. Edited by I. Minis Hays, M. D., Recorder. Philadelphia: William J. Dornan, Printer. 1893.

### Literary Notes.

THE MAGAZINE OF THE FUTURE.—The July *Cosmopolitan* marked the most radical step ever taken in periodical literature. With that issue, the magazine, unchanged in form, in fact one of the best numbers of the *Cosmopolitan* ever issued, was put on sale at twelve and one-half cents per copy—$1.50 a year. The cutting in half of a price already deemed low for an illustrated magazine, is the result of an intention long since formed, to give to the public an illustrated monthly of the very highest class at such a price as must bring it within the reach of all persons of intellectual tastes, however limited their incomes. There are more than 10,000,000 readers in the United States, and less than 800,000 magazines are printed to supply their demands. More than four years have been spent in reaching the organization necessary for the production of the *Cosmopolitan* at this price, a figure hitherto undreamed of by the reading world. Each department of the work has been slowly perfected, until, with the January number of this year, 150,000 copies of the magazine were prepared upon presses and machinery of the most improved form, built with a view to producing the finest results at the very minimum of expense—the only establishment in the world, it is believed, devoted exclusively to the printing of an illustrated monthly magazine. To establish a magazine upon such a basis at the outset was impossible. Only the rapid growth of the *Cosmopolitan's* editions, almost unprecedented in magazine records, has produced the conditions which make this departure from established prices possible. The *Cosmopolitan* promises to make the year 1893 the most brilliant in its history. No other year has seen such an array of distinguished names as will appear on its title page during 1893. De Maupassant, Mark Twain, George Ebers, Valdez, Spielhagen, Francois

